# Sensorimotor Plasticity after Music-Supported Therapy in Chronic Stroke Patients Revealed by Transcranial Magnetic Stimulation

**DOI:** 10.1371/journal.pone.0061883

**Published:** 2013-04-17

**Authors:** Julià L. Amengual, Nuria Rojo, Misericordia Veciana de las Heras, Josep Marco-Pallarés, Jennifer Grau-Sánchez, Sabine Schneider, Lucía Vaquero, Montserrat Juncadella, Jordi Montero, Bahram Mohammadi, Francisco Rubio, Nohora Rueda, Esther Duarte, Carles Grau, Eckart Altenmüller, Thomas F. Münte, Antoni Rodríguez-Fornells

**Affiliations:** 1 Cognition and Brain Plasticity Group [Bellvitge Biomedical Research Institute-] l'Institut d'Investigació Biomèdica de Bellvitge, L'Hospitalet de Llobregat, Barcelona, Spain; 2 Neurodynamic Laboratory, Departament of Psychiatry and Clinical Psychobiology, Universitat de Barcelona, Barcelona, Spain; 3 Department of Basic Psychology, Campus Bellvitge, University of Barcelona, L'Hospitalet de Llobregat, Barcelona, Spain; 4 Hospital Universitari de Bellvitge, Neurology Section, Campus Bellvitge, University of Barcelona - [Bellvitge Biomedical Research Institute-] l'Institut d'Investigació Biomèdica de Bellvitge, L'Hospitalet, Barcelona, Spain; 5 Institute of Music Physiology and Musicians' Medicine, University of Music and Drama Hannover, Hannover, Germany; 6 International Neuroscience Institut Hannover, Hannover, Germany; 7 Department of Physical Medicine and Rehabilitation, Parc de Salut Mar, Hospitals del Mar i de l'Esperança, Barcelona, Spain; 8 Department of Neurology, University of Lübeck, Lübeck, Germany; 9 Catalan Institution for Research and Advanced Studies, Barcelona, Spain; McMaster University, Canada

## Abstract

**Background:**

Several recently developed therapies targeting motor disabilities in stroke sufferers have shown to be more effective than standard neurorehabilitation approaches. In this context, several basic studies demonstrated that music training produces rapid neuroplastic changes in motor-related brain areas. Music-supported therapy has been recently developed as a new motor rehabilitation intervention.

**Methods and Results:**

In order to explore the plasticity effects of music-supported therapy, this therapeutic intervention was applied to twenty chronic stroke patients. Before and after the music-supported therapy, transcranial magnetic stimulation was applied for the assessment of excitability changes in the motor cortex and a 3D movement analyzer was used for the assessment of motor performance parameters such as velocity, acceleration and smoothness in a set of diadochokinetic movement tasks. Our results suggest that the music-supported therapy produces changes in cortical plasticity leading the improvement of the subjects' motor performance.

**Conclusion:**

Our findings represent the first evidence of the neurophysiological changes induced by this therapy in chronic stroke patients, and their link with the amelioration of motor performance. Further studies are needed to confirm our observations.

## Introduction

Stroke is one of the most significant causes of long-term disability in developed countries [1]. Therefore, it is necessary to explore new neurorehabilitation strategies to improve the recovery of limb function and to promote functional cortical reorganization [2]–[4]. The present study aims to investigate the functional changes in the sensorimotor cortex and the associated motor improvements induced by a recently developed therapy based on musical training in chronic stroke patients. To this aim, Transcranial Magnetic Stimulation (TMS) was applied to assess changes on corticomotor representations influenced by rehabilitative interventions, as well as to obtain estimates of the integrity of intracortical and corticospinal excitatory pathways after stroke [5]–[7].

An association between corticomotor excitability changes and recovery of the upper extremity has been well established [8]–[10] although the exact relationship remains unclear. In a pioneer study, Liepert [11] applied TMS to investigate inhibitory and facilitatory neuronal circuits within the primary motor cortex (M1) in chronic stroke patients with moderate hemiparesis who underwent a constraint-induced movement therapy (CIMT). Results revealed an association between the modulation of the inhibitory activity on the affected hemisphere and the improvement of the movement in the affected upper limb. Nonetheless, no conclusive changes were found concerning the excitatory outcomes.

Many studies in humans have demonstrated functional reorganization associated with learning new motor skills [12], [13]. This suggests that the motor cortex might have the potential for functional changes and that training new motor skills might be a responsive therapeutic strategy after brain injury. During the last decade, researchers have paid special attention to the neurophysiological bases of musical processing, especially with respect to the long-lasting effects of fine motor learning [14], [15], auditory motor coupling [16] and the implication of emotion and reward brain networks in these processes [17]. Previous studies have shown rapid functional improvements associated with plastic brain changes due to musical performance, which involved the auditory and integrative auditory-sensorimotor cortices instead of restricted motor cortical areas [18]. Recently, a new motor rehabilitation therapy has been developed based on these investigations: Music-Supported Therapy (MST). This therapy involves repetitive exercises using musical instruments (MIDI piano and electronic drums) in order to train fine and gross motor functions in patients suffering from mild to moderate upper limb paresis after a stroke. Over the past five years, MST has been applied to two large samples of acute stroke patients, reporting relevant improvement in motor performance [19]–[21]. However, it remains unexplored which changes at the neural level were induced by the MST, their correlates with motor performance, and the possible effectiveness of MST in the rehabilitation of chronic stroke patients. Recently, our group reported fMRI and TMS data from one chronic stroke patient that underwent MST, providing first evidence of neuroplastic changes in premotor areas [22].

In the present study, we explored the neurophysiological effects of MST in a group of chronic stroke patients suffering from moderate impairment of upper limb motor function. Before and after the intervention, we assessed several excitatory and inhibitory parameters using TMS, as well as the cortical representation of the first dorsal interosseous (FDI) in both hemispheres. Also, patients were tested with a computerized movement analyzer to evaluate motor performance. This approach allowed us to measure the plastic changes produced by the application of MST and the index of improvement on the affected hand.

## Materials and Methods

The ethical committee from Hospital de Bellvitge gave approval and the experiment was carried out in conformity with the standards set by the Declaration of Helsinki.

### Participants

All participants provided a written consent to participate in this study.

Twenty chronic right-handed stroke patients (15 men, 59.05±9.05 years old) with a slight to moderate hand paresis were recruited at the Hospital Universitari de Bellvitge and Hospital de l'Esperança in Barcelona. Patients were required to have had a single stroke at least 6 months before enrollment and an overall Barthel Index over 50 from a maximum possible score of 100 [23]. History of seizures, marked cognitive impairment, major comorbidity and prior acquisition of musical skills led to patient disqualification from the study. Overall school attendance was 12.4±5.8 years. Table 1 provides additional demographic data.

**Table 1:** Demographic data of each individual of the group of patients.

Subject	Age	Months Since Stroke	MRCS	Lesion Location	Barthel Index
1	42	20	4−	Left Thalamus, Posterior Putamen and Internal Capsule	90
2	65	8	5−	Right Frontal and Temporal Cortex and Striatum	85
3	66	74	4−	Right Internal Capsule and Striatum	95
4	60	11	5−	Left Thalamus	100
5	59	71	4+	Right Frontal, Temporal and Parietal Cortex	95
6	68	10	4+	Right Temporal and Frontal Cortex	75
7	65	14	4+	Left Thalamus	70
8	63	50	5−	Left Subinsular Region and Claustrum	100
9	51	59	4	Left Prerolandic Region	100
10	68	7.5	5−	Right Thalamus	80
11	49	10	4+	Left Thalamus	100
12	71	8	3+	Right Subinsular Region and Frontal Cortex	90
13	66	6	3+	Left Lenticular Nucleus and Internal Capsule	70
14	44	9	5−	Right Pons	100
15	65	6.5	5−	Left Internal Capsule	95
16	60	55	4	Left Putamen and External Capsule	100
17	42	16	4−	Left Frontal and Temporal Cortex	100
18	65	18	5−	Right Caudate and Cerebellum	95
19	57	20	5−	Right Temporal Cortex	95
20	55	13	4+	Left Pons and Occipital Cortex	95

For each participant, the following data was obtained at the time the patient began the study: The age (years), months since the stroke, the Medical Research Council (MRC) score, the location of the lesion and the Barthel Index.

Fourteen right-handed healthy participants (12 men, 56±9.6 years old), within the same age range that the patient group and matched by gender and educational level, were recruited as a control group. Overall school attendance was 11.7±5.1 years. All participants of this group did not report any history of stroke or other neurological disease, seizures, cognitive impairment or major comorbidity.

Right-handedness of all participants in this study was assessed using the Edinburgh handedness inventory [24].

### Music-Supported Therapy

During four weeks, participants from the patient group (PG) received 20 sessions of MST of 30 minutes in duration, administered individually as described by Schenider and colleages [21]. Two different input devices were used: a MIDI-piano to improve fine motor function and an electronic drum pad set to improve gross movements. Each drum pad (numbered 1 to 8, each with 20 cm diameter) was set to produce one of eight piano notes (C D E F G A B C′), rather than drum sounds. Likewise, part of the keyboard of the MIDI-piano was covered such that only eight white keys (C D E F A B C′) could be played by the patient. In this way, fine and gross motor skills could be taxed with two different input devices, while keeping the output constant.

For drum training, participants sat on a chair without armrests in front of the 8 drum pads. Each exercise was first played by the experimenter and was subsequently repeated by the patient. The instructor stood behind the patient and gave support for the affected extremity if necessary. Similarly, patients sat in front of the MIDI-piano with the instructor sitting next to them (on the affected side). Again, an exercise was demonstrated by the instructor first and then repeated by the patient. The therapy comprised a great number of levels with high variability in number of tones, velocity and order of playing (see [20], [21] for details). The difficulty was increased by the experimenter in a stepwise fashion according to the MST manual from playing single tones to playing sequences of notes to playing the beginnings of children's songs.

### Evaluation of motor functions

The evaluation of the motor functions was conducted using two different instruments.

First, we used the Action Research Arm Test (ARAT) [25] to assess the pertinent functions of the upper extremities within four subtests: grasp, grip, pinch and gross movement. The maximum possible score in this test is 57.

Second, we used a computerized movement analysis system (CMS 30 P, Zebris, Isny, Germany) to evaluate quantitatively the efficacy of the MST on the motor behavior. This system is based on the continuous recording of the position of ultrasonic markers placed on the upper limb. The spatial coordinates of these markers were sampled with a frequency of 66 Hz and a spatial resolution of 0.1 mm. Continuous calculation of the three dimensional position of each sender was carried out using commercially obtainable software (WinData 2.19.3×, Fa, Zebris, Isny, Germany).

Three self-paced diadochokinetic movements of the upper limb were tested on each hand: whole hand tapping (HT), index finger tapping (FT), and forearm pronation and supination (PS). For details of movement registration and position of the markers see [26]. Participants were instructed to move as fast as possible during the movement registration. Three measures were assessed for each task:

- Frequency (FREQ), defined as the number of cycles performed per second.
- Average maximum velocity (VMAX) in millimeters per second
- Number of Inversions of Velocity (NIV) per movement segment, as a measure of smoothness of the movement. Inversions with amplitudes less than 3% of maximal velocity were excluded. This measure could reach 1 as a best value.

Data analysis was performed as described by Schneider and colleagues [21] using commercially obtainable software (3DAWin-Version 1.2, C-Marquardt, Munich, Germany). As suggested by Hermsdörfer and colleagues [26], both the first and the last movement cycles were omitted to exclude artifacts due to movement onset or fatigue. Five movement cycles were selected by marking the movement onset of the second and the offset of the sixth cycle by visual inspection. In this way, three measurements were conducted per task. Subsequently, a segment analysis was made for the selected movement cycles. Data were averaged over the five cycles and three repetitions to obtain one measurement for each participant. Finally, these data were entered into the statistical analysis, details of which are explained in the Analysis section.

### Transcranial Magnetic Stimulation

Single pulse TMS was performed using a 70 mm figure-of-eight coil attached to a Magstim Rapid 2 (Magstim Company, Carmathenshire, Wales). For each pulse, we collected electromyographic (EMG) activity from the contralateral FDI muscle for a total of 700 ms including a 100 ms pre-stimulus window (Medelec Synergy, Oxford Instruments, Pleasantville, NY, USA) using surface Ag/AgCl disk electrodes in a belly-tendon montage. The EMG signal was sampled at 5 KHz and band-pass filtered at 1–1000 Hz. Data were stored and exported for off-line analysis using specialized software (Matlab, Mathworks, Natick, MA, USA). Participants were fitted with an elastane cap on which a 10×10 cm grid centered on the vertex (Cz position of the international 10/20 EEG positioning system) was drawn to allow simple identification of stimulation coordinates for sites separated by 1 cm each. For details of the grid composition see [22]. To reduce inter-session variability, a single cap was used per participant in both evaluations. The TMS coil was placed tangentially to the corresponding grid location, with the handle pointing backwards (in a lateral to medial and caudal to rostral position) ∼45° lateral from the midline. For each participant, the following parameters were assessed for each hemisphere: The hot spot, the resting and active motor threshold (RMT and AMT), the peak-to-peak motor evoked potential (MEP), the cortical silent period (CSP), the slope of the recruitment curve (RC), the area and the center of gravity (CoG) of the topographic representation of the motor map. The hotspot site was identified as the grid position of maximum response to the magnetic stimulation [27]; the RMT was defined as the stimulus intensity needed to elicit a MEP of 50 µV with the FDI at rest [27]; the AMT was defined as the stimulus intensity needed to elicit a MEP of 200 µV during a voluntary contraction of the FDI [28]; the CSP was considered as the length of the EMG signal corresponding to the transient suppression of the ongoing motor activity after TMS-evoked muscle activation during voluntary FDI contraction [29], [30]. For its assessment, fifteen pulses were given at the hotspot, each at stimulation of 150% of the RMT; the slope of the RC was generated by stimulation over the hotspot with five trials each at stimulation intensities of 90% to 160% of the RMT pseudo randomly [11]; the peak-to-peak motor evoked potential (MEP) amplitude was obtained by stimulation over the hotspot with ten trials each at stimulation of 130% of the RMT; the topographic representation of the motor map was calculated as the mean of the peak-to-peak MEP amplitude obtained by stimulation over all active sites of the scalp with five trials each at stimulation of 120% of the RMT. The area of the map was calculated by counting the number of active locations. Coordinates of the center of gravity of the map (CoG_x_ and CoG_y_) were calculated as a weighted sum of the amplitudes obtained in the map [31], [32].

## Analysis

All the experimental procedures and evaluations here described were performed and analyzed in the Hospital de Bellvitge. All participants from the PG were examined before and after the application of the MST. Participants from the control group (CG) were examined by the same procedures as patients (PG), but they did not undergo any MST session and 30 to 40 days elapsed between evaluations. All TMS parameters and evaluation of the motor performance were examined twice, prior to the beginning of the MST and within the following week after the end of the therapy. The same stimulation intensity was used in both evaluations of a patient or control participant. For the calculation of peak-to-peak MEP amplitude, RC, CSP and motor map measures we used the same intensity in both evaluations, relative to the RMT obtained at the first evaluation. When corresponding stimulation intensities exceeded the maximal stimulation output (MSO), the 100% of stimulus intensity was used. We applied all subtests of the ARAT test to participants, but only the global score of the test was considered for the analysis.

Statistical analysis was performed using SPSS 17 (SPSS, Chicago, Illinois). For all measures, we compared the affected hemisphere of each patient to the corresponding side of the corresponding matched control participant. The same procedure was used to compare the unaffected hemisphere between groups. To measure the effect of therapy, a mixed 2×2 repeated measures analysis of variance (ANOVA) with factors Group (PG and CG) and Time (pre- and post-therapy evaluations) was used for each hemisphere. Bonferroni corrected paired t-tests were used for post-hoc comparisons. Correlation analyses between NIV of hand tapping and (i) MEP and (ii) mediolateral displacement of the center of gravity were done with Pearson's coefficient.

## Results

### Motor function test

Each participant from the PG was able to perform all movement tasks correctly with the unaffected hand. For the ARAT test, each participant from the PG performed all subtests with the affected hand. However, three of them had difficulties performing correctly the forearm pronation and supination with the affected hand, and two more patients were also unable to perform correctly the hand tapping task with the affected hand. As a result of this, these patients were removed for the analysis of dependent variables involving these movements for the affected and the unaffected hands. All participants from the CG were able to perform all motor function tests correctly.

**Figure 1:**
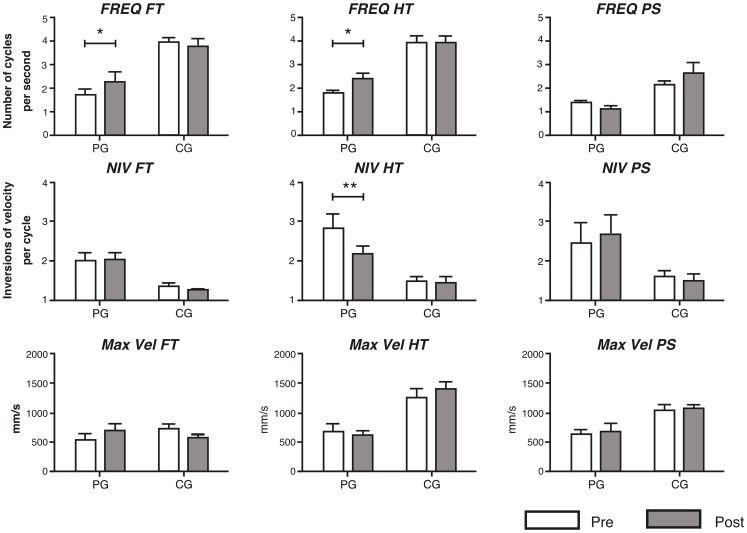
Summary of the kinematic parameter for the patients' group (PG) and control group (CG). Bars represent the mean of frequency (FREQ), number of inversions of velocity (NIV) and the average maximum velocity (Max Vel) for finger tapping (FT), hand tapping (HT) and forearm pronation-supination (PS). Error bars represent the standard deviation of the mean (SEM). Significant differences after training in the MG are indicated (* p<.05, ** p<.01).

**Figure 2:**
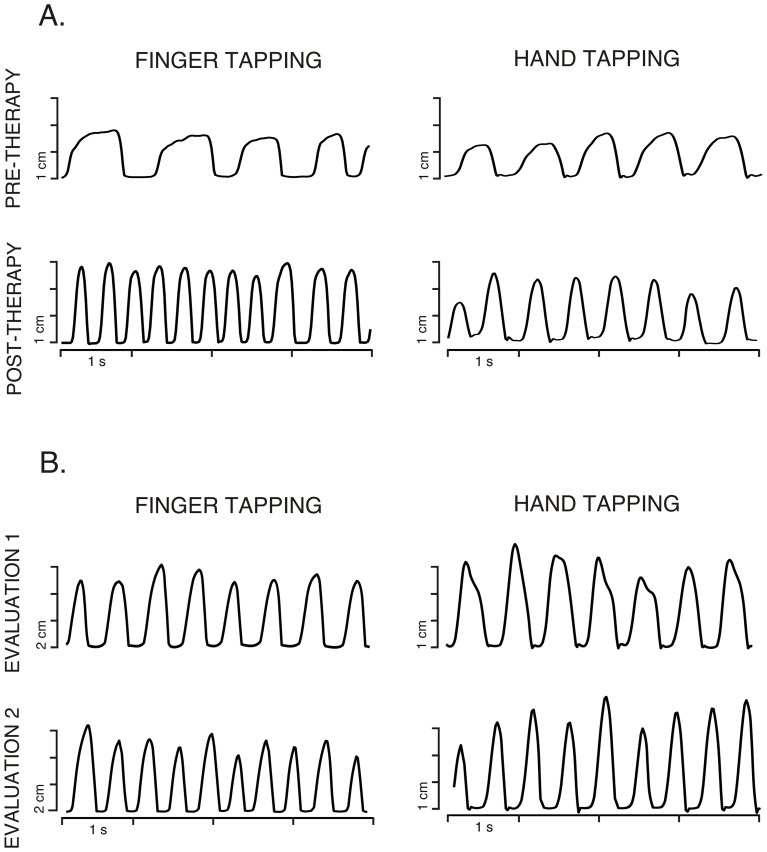
Example of performance of fast diadochokinetic finger. Detail of the signal recorded from one marker with the 3D movement-analyzer during finger and hand tapping movements of the affected hand of one representative patient (A) and one control subject (B). The marker was attached on the index finger (finger tapping) and from methacarpophalangeal joint (hand tapping). Time courses of the displacement in cm measured in both evaluations are displayed.

All the results for the different ANOVAs performed on each parameter for the computerized movement analysis are shown in the table 2. Participants from the CG did not show any significant changes between sessions in any kinematic parameter [|*t*(13)|<1.5]. We did not find any significant Group×Time interaction in any analyzed parameter on the unaffected hemisphere in subjects of the PG. On the affected hemisphere, we found significant Group×Time interactions in the NIV of hand tapping, FREQ of pronation-supination, and a marginal interaction in FREQ of finger tapping (see table 2). Subjects from the PG showed evident increases in the frequency of affected finger tapping [*t*(19) = −2.43, *p*<.05], and a large decrease on the NIV for the affected hand tapping task [*t*(17) = 3.84, *p*<.01], between the pre- and post-therapy evaluations (see Figures 1 and 2).

**Table 2:** Results of ANOVA analysis of each dependent variable concerning diadochokinetic movements performed with the contralateral hand.

	AFFECTED HEMISPHERE		UNAFFECTED HEMISPHERE	
	Time-Point (Pre/Post)	Group×Time Point	Time-Point (Pre/Post)	Group×Time Point
FREQ FT (cycle/s)	F(1,32) = 2.09 n.s.	F(1,32) = 2.85+	F(1,32) = 2.9+.	F(1,32) = 0.002 n.s.
VMAX FT (deg/s)	F(1,32) = 0.001 n.s.	F(1,32) = 2.7+	F(1,32) = 0.9 n.s.	F(1,32) = 0.7 n.s.
NIV FT	F(1,32) = 0.08 n.s.	F(1,32) = 0.11 n.s.	F(1,32) = 3.2 n.s.	F(1,32) = 1.3 n.s.
FREQ HT (cycle/s)	F(1,30) = 1.7 n.s.	F(1,30) = 1.68 n.s.	F(1,30) = 0.1 n.s.	F(1,30) = 0.3 n.s.
VMAX HT (deg/s)	F(1,30) = 0.09 n.s.	F(1,30) = 0.7 n.s.	F(1,30) = 0.7 n.s.	F(1,30) = 0.6 n.s.
NIV HT	F(1,30) = 6.9 **	F(1,30) = 5.05 *	F(1,30) = 2.1 n.s.	F(1,30) = 0.5 n.s.
FREQ PS (cycle/s)	F(1,27) = 2.6 n.s.	F(1,27) = 4.46 *	F(1,27) = 1.05 n.s.	F(1,27) = 2.3 n.s.
VMAX PS (deg/s)	F(1,27) = 0.3 n.s.	F(1,27) = 0.053 n.s.	F(1,27) = 0.04 n.s.	F(1,27) = 0.002 n.s.
NIV PS	F(1,27) = 0.04 n.s.	F(1,27) = 0.002 n.s.	F(1,27) = 0.008 n.s.	F(1,27) = 0.23 n.s.

Units of each dependent variable are also shown. Notes: FREQ = frequency; VMAX = maximal velocity; NIV = number of inversion of velocity; FT = finger tapping; HT = hand tapping; PS = pronation-supination; deg = degree; s = second.

Prior to the therapy, participants from the PG had a global ARAT score (M ± STD) of 42.19±15.27 for the affected hand. After the application of the therapy, the ARAT scored 46.6±13.89. Such an increase of the ARAT score was significant [*t*(19) = −3.84, *p* = .001]. In both evaluations, the unaffected hand scored the maximum value of the test (57). When applied to participants from the CG, the test scored maximum for both hands in both evaluations.

### Transcranial Magnetic Stimulation

One patient was not included in the TMS protocol due to a previous cranial surgery and three more patients disagreed to be included in the TMS protocol. Two more patients missed the second evaluation for health issues not related to the protocol. Finally one subject showed a resting motor threshold of about 100%, which was considered too high to follow the whole TMS protocol; hence, only resting and active motor thresholds from this patient were considered as dependent variables. Therefore, fourteen patients succeed in taking part in both sessions of the whole TMS protocol. All participants from the CG were examined twice successfully. No participant reported any side effects after the application of TMS in either of the sessions.

**Figure 3:**
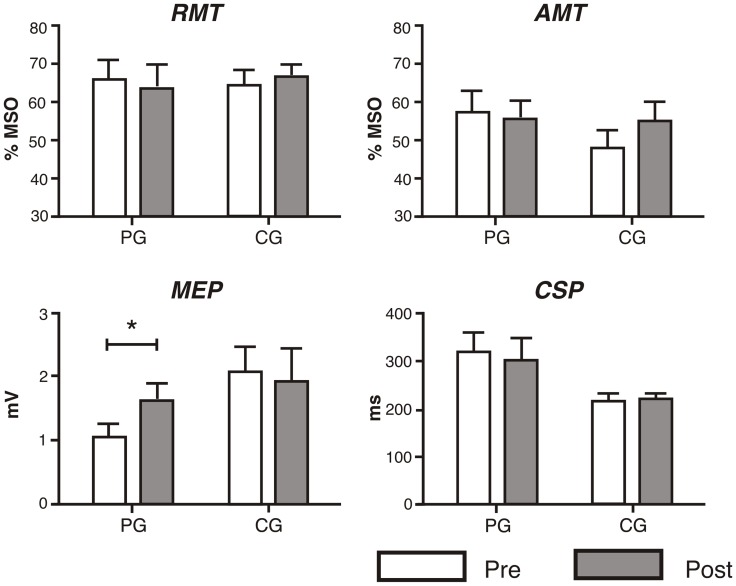
Summary of the results of the TMS study. Bars represent the mean of the motor evoked potential (MEP) amplitude, length of the cortical silent period (CSP), resting motor thresholds (RMT) and active motor thresholds (AMT) of the affected hemispheres for PG and CG. Error bars represent the standard deviation of the mean (SEM). Measurements at the evaluation 1 are colored in white, and measures at the evaluation 2 are colored in gray. An increase of the MEP amplitude on the affected hemisphere in PG can be observed (* p<. 05).

**Figure 4:**
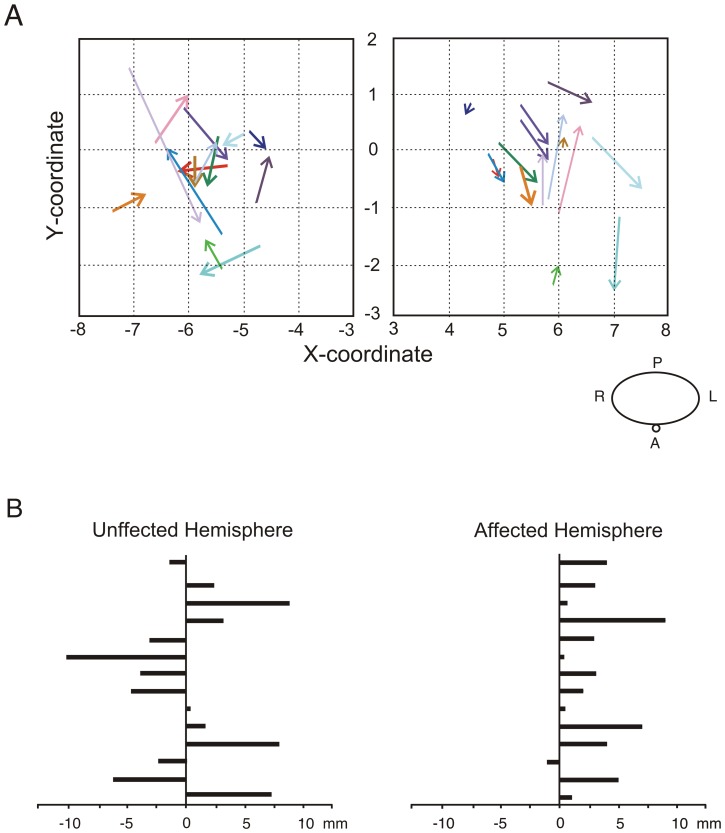
Summary of the plastic changes observed with the cortical maps. (A) Displacement of the center of gravity (CoG) across time for the affected and unaffected hemisphere in PG. The origin of each arrow represents the baseline coordinates of the CoG. Arrowheads represent the position of the CoG at evaluation 2. Each arrow is colored differently, corresponding to each subject from PG. (B) Displacement of the medial coordinate of the center of gravity (CoGx) of motor mapping representation through time. In the affected hemisphere (left), almost all patients showed a displacement on the mediolateral edge of the CoG to more temporal regions.

Results for the main TMS parameters are shown in figure 3. A list of the results of the conducted ANOVAs is shown in the table 3. We did not find any Group×Time interaction in any measured parameter on the unaffected hemisphere. In the affected hemisphere, however, we found a marginal Group×Time interaction in MEP amplitude and a significant interaction in the displacement of the center of gravity through the mediolateral axis. Post hoc comparisons revealed that subjects from the PG presented a significant shift of CoG_x_ away from the vertex [*t*(13) = −3.84, *p*<.01], as is shown in Figure 3. Furthermore, as illustrated in Figure 4, we found a significant increase of MEP amplitude on the affected hemisphere [*t*(13) = −2.6, *p* = .02]. In the unaffected side, MEP amplitude did not change over time; neither did the rest of electrophysiological measurements. Subjects from the CG did not show any change over time in any electrophysiological parameter [|*t*(13)|<1.5].

**Table 3:** Results of ANOVA analysis of each dependent variable concerning electrophysiological measurements obtained after the stimulation of each hemisphere (affected and unaffected) (* *p*<.05; ^+^.05<*p*<.1; n.s. not significant).

	AFFECTED HEMISPHERE		UNAFFECTED HEMISPHERE	
	Time-Point (Pre/Post)	Group×Time Point	Time-Point (Pre/Post)	Group×Time Point
RMT (% stimulator)	F(1,27) = 0.25 n.s.	F(1,27) = 2.63 n.s.	F(1,27) = 0.44 n.s.	F(1,27) = 2.67 n.s.
AMT (% stimulator)	F(1,27) = 0.49 n.s.	F(1,27) = 2.27 n.s.	F(1,27) = 2.56 n.s.	F(1,27) = 0.88 n.s.
MEP amplitude (µV)	F(1,26) = 1.32 n.s	F(1,26) = 3.53+	F(1,27) = 6.43 *	F(1,27) = 0.1 n.s.
CoGx	F(1,26) = 1.2 n.s.	F(1,26) = 7.14 *	F(1,27) = 0.1 n.s.	F(1,27) = 0.14 n.s.
CoGy	F(1,26) = 0.09 n.s.	F(1,26) = 0.001 n.s.	F(1,27) = 0.084 n.s.	F(1,27) = 1.51 n.s.
CSP (ms)	F(1,26) = 0.43 n.s.	F(1,26) = 0.49 n.s.	F(1,27) = 0.27 n.s.	F(1,27) = 0.91 n.s.
Mapping Area (cm^2^)	F(1,26) = 0.086 n.s.	F(1,26) = 0.3 n.s.	F(1,27) = 2.09 n.s.	F(1,27) = 0.8 n.s.
Mapping Volume	F(1,26) = 0.23 n.s.	F(1,26) = 0.47 n.s.	F(1,27) = 0.6 n.s.	F(1,27) = 1.31 n.s.
Recruitment Curve Slope	F(1,26) = 0.12 n.s.	F(1,26) = 0.02 n.s.	F(1,27) = 0.21 n.s.	F(1,27) = 0.44 n.s.

Notes: MEP = peak-to-peak amplitude of the motor evoked potential; RMT = resting motor threshold; AMT = active motor threshold; CoG_x_ = mediolateral coordinate of the center of gravity; CoG_y_ = anteroposterior coordinate of the center of gravity; CSP = cortical silent period; µV = microvolts; ms = milliseconds; cm^2^ = squared centimeters.

**Figure 5:**
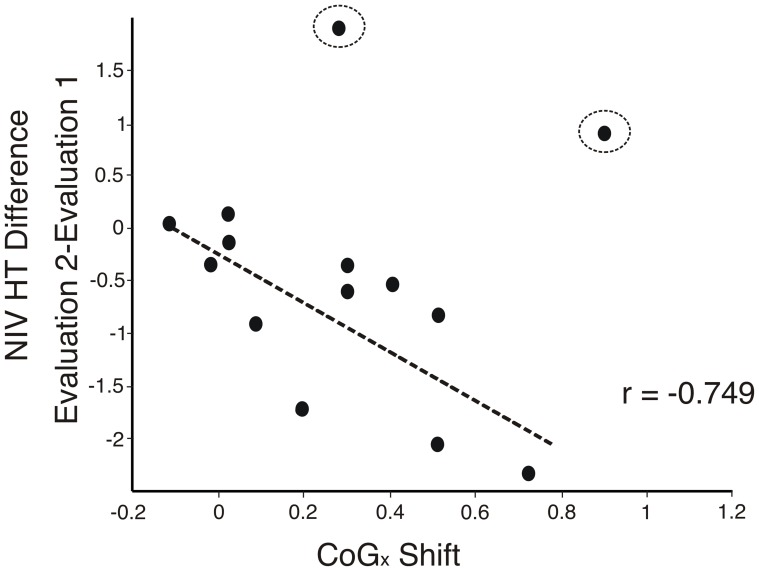
Correlation between changes on the position of the CoG in the mediolateral edge and changes on the number of inversion of velocity (NIV) for hand tapping (HT) from subjects in PG. Rounded points were considered outlier values after corresponding test and were excluded from the sample.

In addition, a significant correlation was found in the PG between the improvement of the affected hand tapping task, in terms of the NIV index, and the magnitude of the shift of the CoG_x_ (*r* = −.75, *p* = .005) of the affected hemisphere (Figure 5). No correlation was found between the increase of MEP amplitude and the improvement of the motor skills (*p*>.4).

## Discussion

In the present study, we have observed significant motor gains accompanied by plastic changes in chronic stroke patients who were tested before and after 20 sessions of music supported therapy (MST). Of note, we found increased motor cortex excitability in the patients' affected hemisphere after training, an association between changes in the motor cortex representation on the injured hemisphere and improved performance of diadochokinetic movements with the affected upper limb. These results suggest that MST can drive task-dependent cortical reorganization in stroke patients in the chronic stage.

Participants from the PG showed clear improvements regarding the range of possible movements (ARAT test score), frequency for finger tapping and the quality of movement (NIV). Similar results were obtained previously in two samples of subacute stroke patients [19], [21]. Our data suggests that the MST caused motor improvement in our group of chronic stroke patients that are generalized for real-word situations, in agreement with Schneider and colleagues [21].

In addition to the motor improvement of the affected upper limb, we found changes on the excitability over the motor cortical areas of the affected hemisphere, as well as a shift of the cortical representation of a muscle by mapping the motor cortex with TMS. The increase in excitability on the corticospinal tract, evidenced by an enhanced MEP-amplitude, might be a consequence of the MST intervention, as no changes were found in a matched control group. However, no changes in motor thresholds were found after the intervention. It has been suggested that changes in the MEP amplitude represent the modulation of the strength of synaptic transmission along on the corticospinal tract and it has been termed as long-term potentiation (LTP) and long-term depression (LTD) –like plasticity [33]. In contrast, motor thresholds measured by TMS are thought to reflect the axonal membrane excitability, since they are influenced by drugs that block calcium voltage-gated ion channels [34]. Consequently, our results suggest that the increase of excitability demonstrated by the enlargement of MEP amplitudes might be explained by an increase of the strength of synaptic transmission rather than a decrease of the threshold of membrane potential. These results are coherent with previous studies demonstrating the effects on the modulation of the strength of synaptic plasticity within the motor cortex during the learning of new skills [12], [13], [32]. Nonetheless, Liepert [11] failed to find an increase of the MEP amplitude on the affected hemisphere after application of CIMT. We did not find a correlation between the increase of MEP amplitude and improvement of motor skills. Nor did we observe significant changes on the linear RC slope between sessions, which is an indicator of the excitability distribution, although its role as an indicator of plasticity is still unclear [35].

Importantly, in the present study we used the CoG of the cortical representation of the FDI as a measure of possible changes in the somatotopy of the motor cortical projections. As reported by Platz et al. [8], we have considered both coordinates of CoG as a measure of localization and their shift across sessions as an index of a change in the motor map. We found that the mediolateral coordinate (CoG_x_) moved away from the vertex on the patients' affected hemisphere after application of MST. Displacement of the motor representation after a rehabilitative intervention has been reported previously ([5], [9] see however [1]). This shift of the center of gravity might indicate the recruitment of additional neural populations which are next to the boundaries of the previous motor map [36]. In addition, the changes in motor cortex representation were associated with an improved motor performance: patients with a greater improvement in smoothness on the performance of movements, quantified by the reduction in the NIV index during hand tapping, presented a greater displacement of CoG_x_ coordinate. These results are in agreement with previous reports [8], [36] establishing an association between the displacement of the CoG across sessions and the improvement of the performance in behavioral motor tests in acute stroke patients. Thus, we could argue in the same way as Platz and colleagues [8], suggesting that the recruitment of adjacent brain areas within the motor cortex could be related with the degree of motor recovery in chronic stroke patients. This finding might demonstrate that the audio-motor coupling enhances reorganization within the affected motor cortex during the chronic phase of stroke.

A remaining open question concerns whether changes on cortical excitability and mechanisms of plastic reorganization might be a consequence of auditory-motor integration. As suggested in different studies, MST has been devoted to potentiate residual learning abilities in acute and chronic patients through indirect but intact brain pathways engaged by music performance [19], [21], [22]. In addition, our data revealed an overall effect of the MEP also in the unaffected hemisphere, suggesting the recruitment of additional contralesional corticospinal fibers. Training-induced motor recovery can be associated with increased activity of the premotor cortex, as well as other cortical and cerebellar areas [37], [38] and it has been suggested that repetition of movements is key to the plastic changes. In a chronic stroke patient who underwent MST we found an increase in BOLD signal in premotor areas of the affected hemisphere when she listened to a set of trained versus untrained melodies [22]. In a recent preliminary fMRI study we also showed increased connectivity between auditory and premotor regions after MST in three chronic stroke patients [39]. These results suggest that for MST audio-motor coupling can be an additional mechanism contributing to neuroplastic changes leading the improvement of the subjects' motor function. This idea is also in agreement with the essential role of audio-motor interactions in music processing [18] and the potential increase of plasticity when using multimodal learning paradigms (see [40]; see for recent reviews [41]–[43]). Interestingly, as in the previous studies (see [21]), we evaluated motor function using diadochokinetic movements not specifically involving the movements requested for music playing during the MST. Thus, while audio-motor integration might help patients to improve during practice, these improvements are then generalized to other situations unrelated to the trained task. However, further research is requested in order to clearly disentangle the specific role of audio-motor integration in the success of the MST.

A number of limitations of the current study should be considered. Although the number of patients is relatively large compared to similar intervention studies, there was a considerable variability in terms of type and location of the lesion, which might have affected the clinical outcome and the electrophysiological measurements. Instead of a clinical control group involved in another kind of therapy, a matched healthy control group was used. Similar studies have reported accurate crossover designs, such as multiple baseline evaluations [44] or including control groups consisting of participants with the same clinical condition but with different or no intervention [20]. Thus, we cannot directly address questions of differential efficacy of MST compared to other interventions, e.g. CIMT in chronic patients, limiting the conclusions of the present study. Note, however, that Schneider and colleagues [20] reported a comparison of MST and CIMT in a sample of acute stroke patients and found effects of MST to be stronger than that of CIMT applied in the same setting and with the same duration. Obviously, a comparison of MST and CIMT in chronic patients would also be desirable. As our patients were in the chronic phase, spontaneous improvements were not to be expected [44]. An important caveat in the present study has been the inclusion of patients affected in the right hemisphere and affected in the left hemisphere. Since all patients from this sample are right-handed, it could be arguable that patients with lesions in the left hemisphere might have developed compensatory motor programs for increasing right-hand utilization when compared with patients with right-hemisphere lesions. Although controlling for this effect might be important in future studies, it was not feasible in the present study due to the small sample size. An additional issue that might limit the interpretation of the results concerns the test-retest reliability of the parameters obtained using the TMS. However, results from prior studies suggest that the reliability of the assessments obtained after magnetic stimulation allows assuming that differences are an outcome of the intervention rather a retest reliability phenomena [1].

In conclusion, we have found that MST induces motor improvements in chronic stroke patients that were accompanied by an increase of the excitability of the corticospinal tract and a modification of the motor cortical representation. We hypothesize that these changes reflect a training-dependent plasticity effect driven by the acquisition of new motor skills with the affected upper limb, leading to a recruitment of bounded brain areas within the motor cortex. These described findings support that the audio motor coupling might enhance plasticity within the affected motor cortex when applied to stroke patients in the chronic phase. However, more studies with different designs are needed to confirm our observations.
